# Slipped capital femoral epiphysis and hypothyroidism in a young adult: a case report

**DOI:** 10.1186/1752-1947-8-336

**Published:** 2014-10-10

**Authors:** Danao Marquez, Eric Harb, Hugo Vilchis

**Affiliations:** Departamento de Ortopedia, Hospital General “Dr. Manuel Gea González”, Secretaria de Salud México, 4800 Calzada de Tlalpan, Seccion XVI, Tlalpan, DF 14080 Mexico

**Keywords:** Adult, Capital, Epiphysis, Female, Femoral, Hypothyroidism, Slipped

## Abstract

**Introduction:**

Slipped capital femoral epiphysis is the most common hip disorder affecting the adolescent population, usually individuals between 8 and 15 years old. However, there are few case reports of older patients in the literature to date. It is believed that the etiology is multifactorial and may include obesity, trauma and, less frequently, endocrine pathologies comprising hypothyroidism, hypogonadism and panhypopituitarism.

**Case presentation:**

We present the case of a 28-year-old Latino woman diagnosed with hypothyroidism secondary to arachnoidocele associated with skeletal immaturity and slipped capital femoral epiphysis.

**Conclusions:**

It is important to conduct clinical and radiographic studies in these patients to rule out endocrine pathologies, especially hypothyroidism in those of sexual maturity.

**Electronic supplementary material:**

The online version of this article (doi:10.1186/1752-1947-8-336) contains supplementary material, which is available to authorized users.

## Introduction

Slipped capital femoral epiphysis (SCFE) is the most common hip disorder affecting the adolescent population, usually between 8 and 15 years old [1, 2]. However, there are few case reports in the literature describing this condition in older patients. It is believed that the etiology is multifactorial and may include obesity, trauma and, less frequently, endocrine pathologies comprising hypothyroidism, hypogonadism and panhypopituitarism [3].

In this report, we present a case of a 28-year-old woman with secondary hypothyroidism, as well as a literature review.

## Case presentation

**Figure 1:**
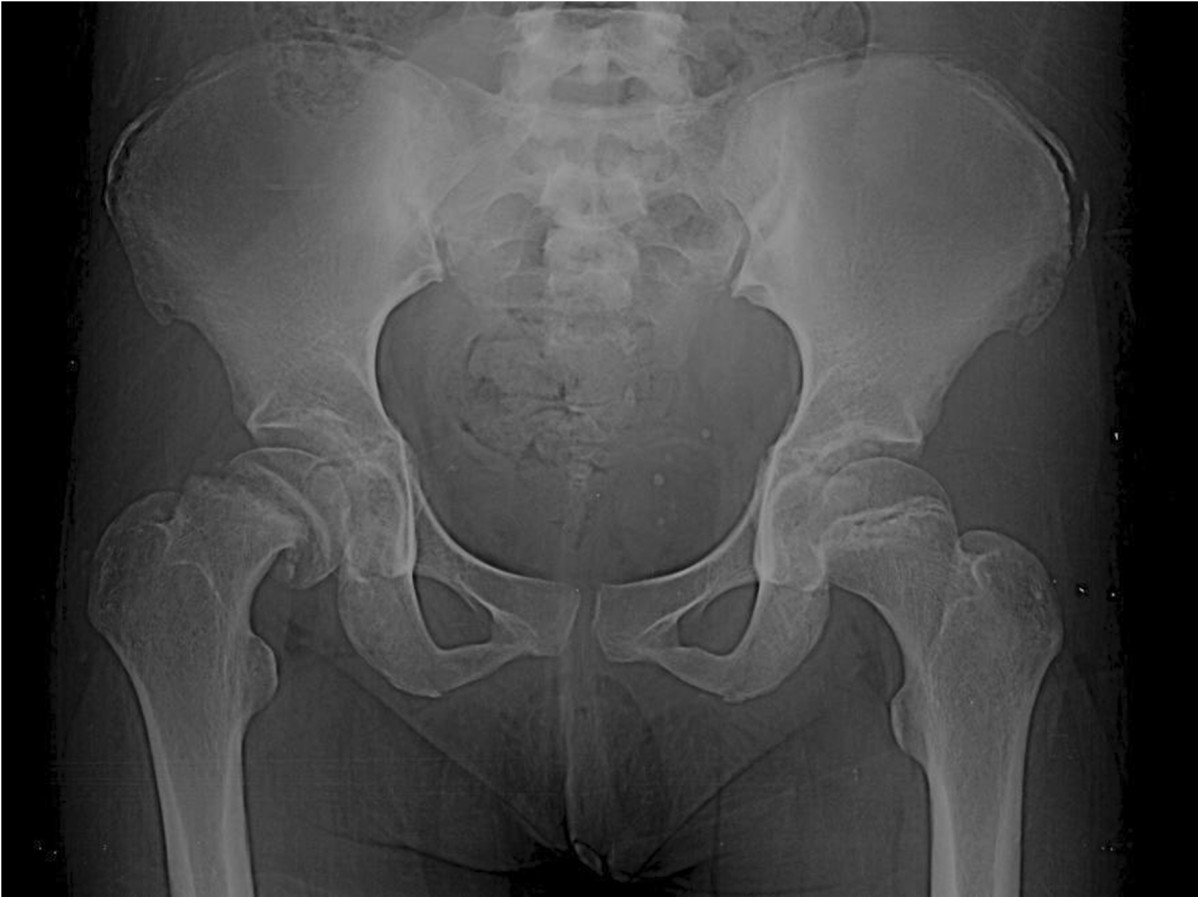
**Pre-operative radiograph showing moderate-grade slipped capital femoral epiphysis and Risser grade IV calcification.**

A 28-year-old Latino woman fell from her own height 4 weeks prior to presentation at our hospital. The fall had caused a direct contusion in her right hip that led to severe pain and functional disability. She had initially been evaluated at the general hospital in her hometown, where she received conservative treatment that included analgesics and rest. She had partial improvement; nevertheless, when she began returning to normal activities, she did not tolerate the pain and attended our unit for care.At admission, she had a medical history of gesta 1, para 1, with no other pathologies referred. The physical examination at presentation revealed short stature, proper development of sexual characteristics (Tanner 4), right pelvic limb with an attitude of flexion to 30° and external rotation, inability to bear weight, range of motion of the right hip flexion to 40°, extension to 10°, abduction to 20°, adduction to 10°, external rotation to 40°, internal rotation to 0°, muscle strength 5/5 and unaltered sensitivity and reflexes. X-rays showed a moderate SCFE (Southwick scale) with a 52° angle, as well as the presence of an opening of the left femoral head physis and Risser grade IV calcification (Figure 1).

### Diagnosis

**Figure 2:**
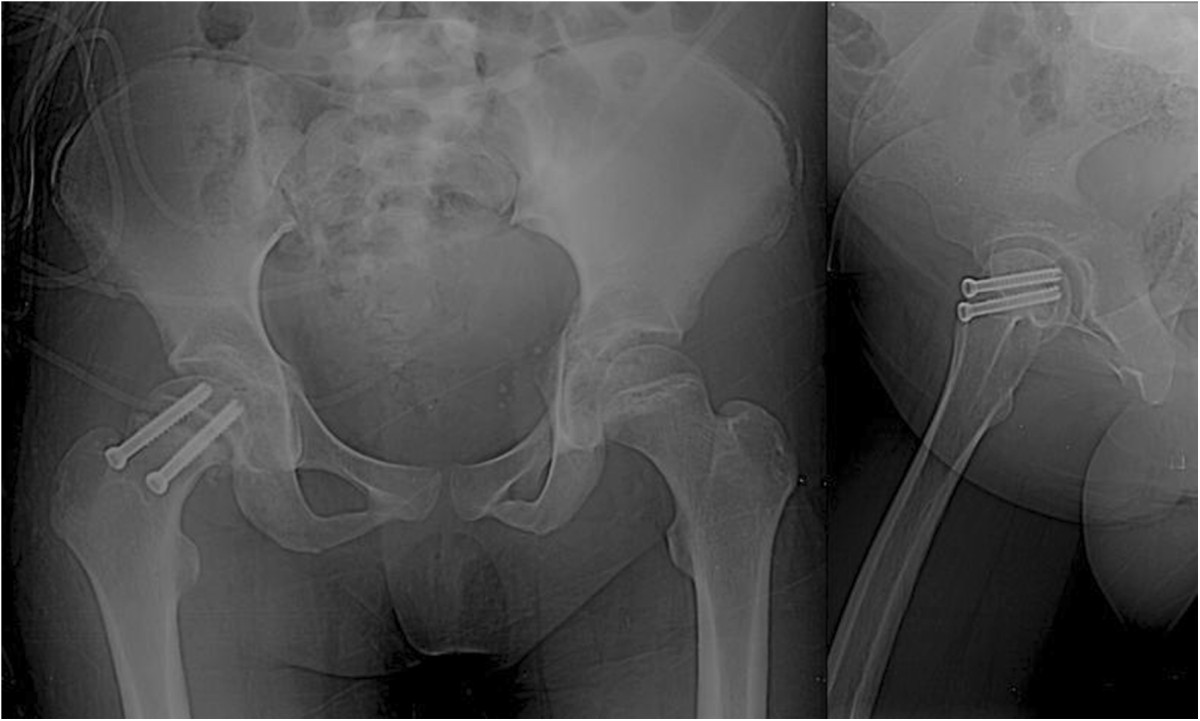
**Post-operative radiograph of the patient’s right hip, where reduction by two cannulated 4.0 screws at a 6° Southwick angle can be seen.**

**Figure 3:**
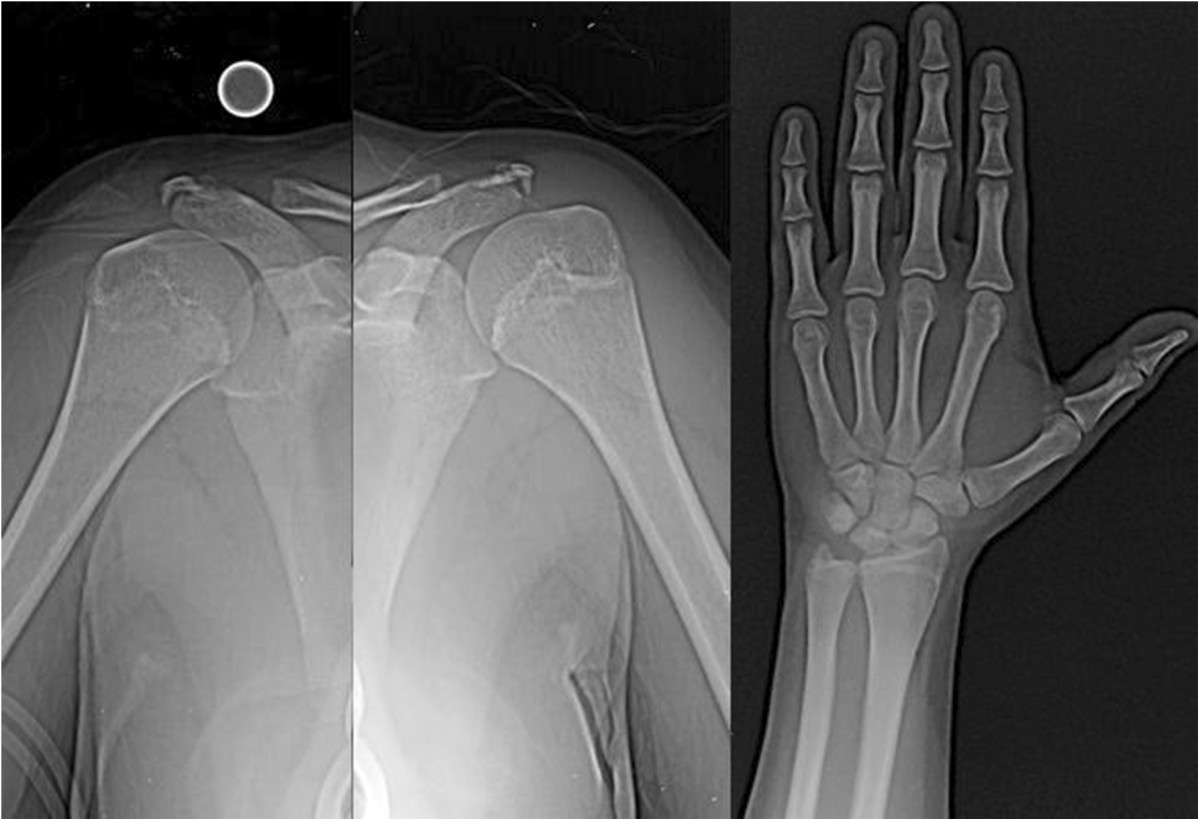
**Anteroposterior radiographs of both shoulders and the right wrist, where the presence of open physis can be observed.**

On the basis of our observations during the patient’s physical examination, we made a diagnosis of moderate unstable SCFE on the right side and delay in physeal closure (without etiology at the time).Closed reduction and internal fixation with two cannulated 4.0 screws at a 6° Southwick angle were performed (Figure 2). Bone series were performed, which showed open physis in the proximal humerus and distal radius (Figure 3).

### Hormone profile

**Figure 4:**
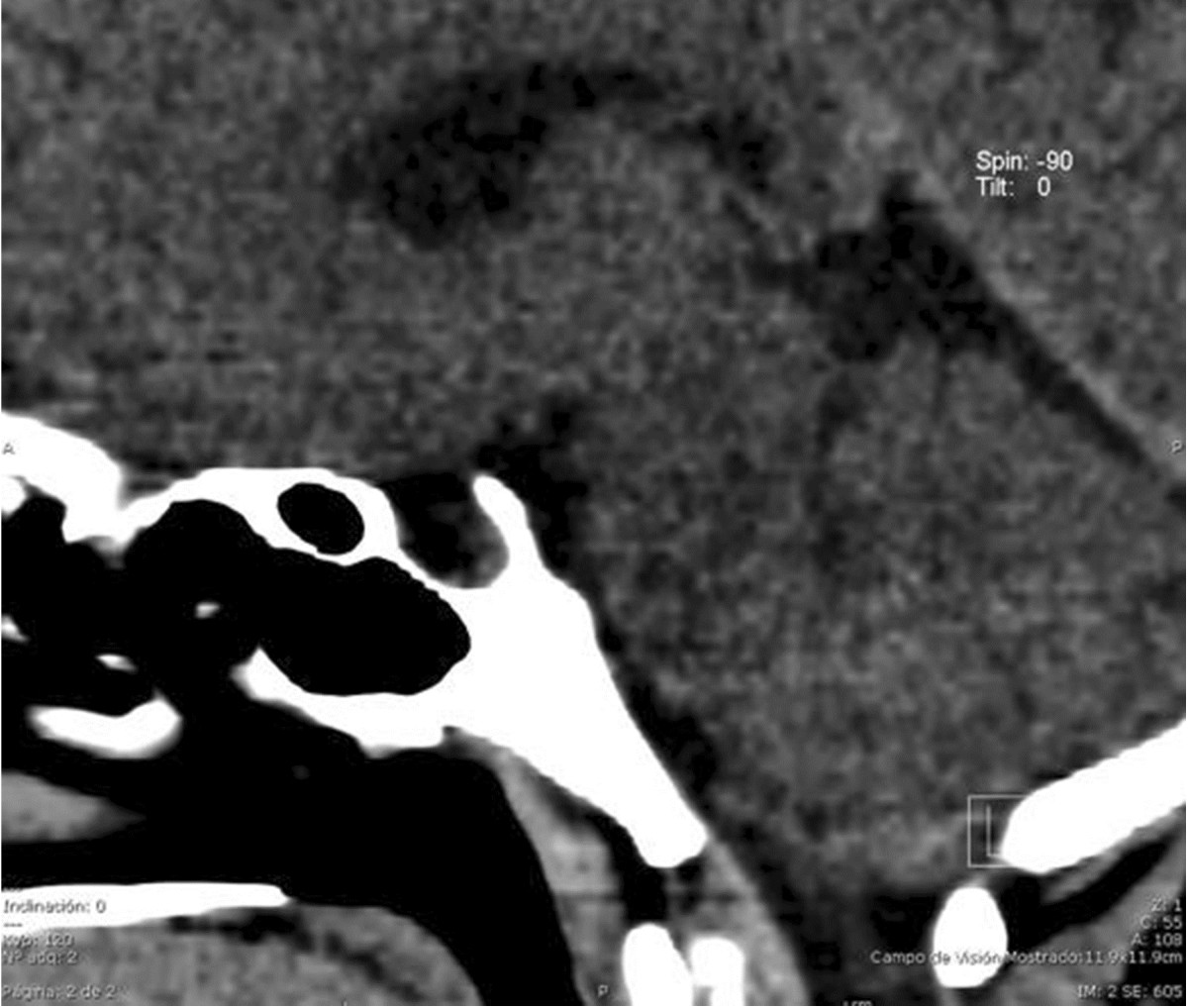
**Sagittal computed tomography scan of the sella.** Arachnoidocele compression of the pituitary gland in 70% is shown.

**Figure 5:**
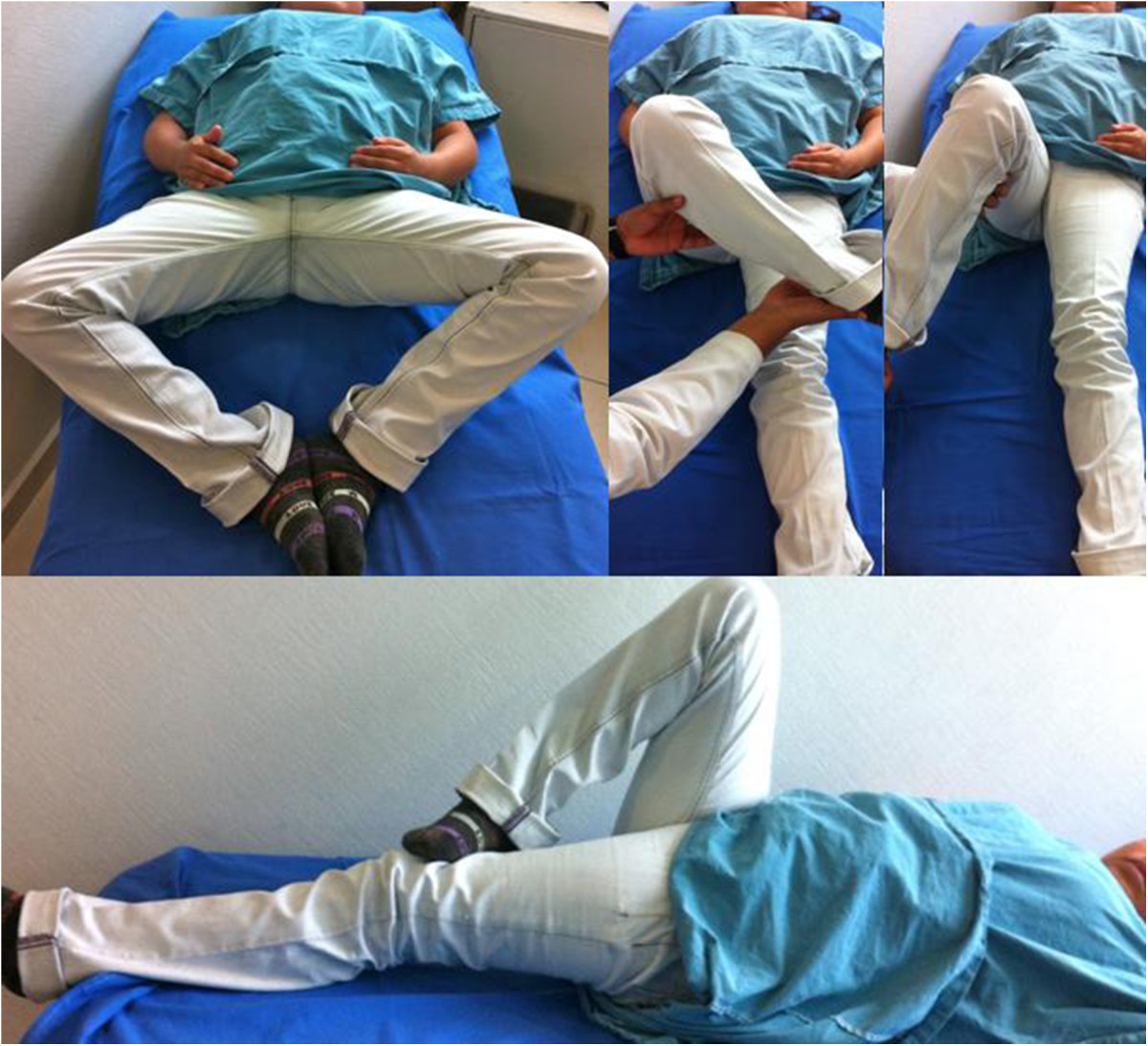
**Photographs of the patient taken at the 4-month follow-up examination.** At 4 months after surgery, the patient’s hip mobility had improved.

**Figure 6:**
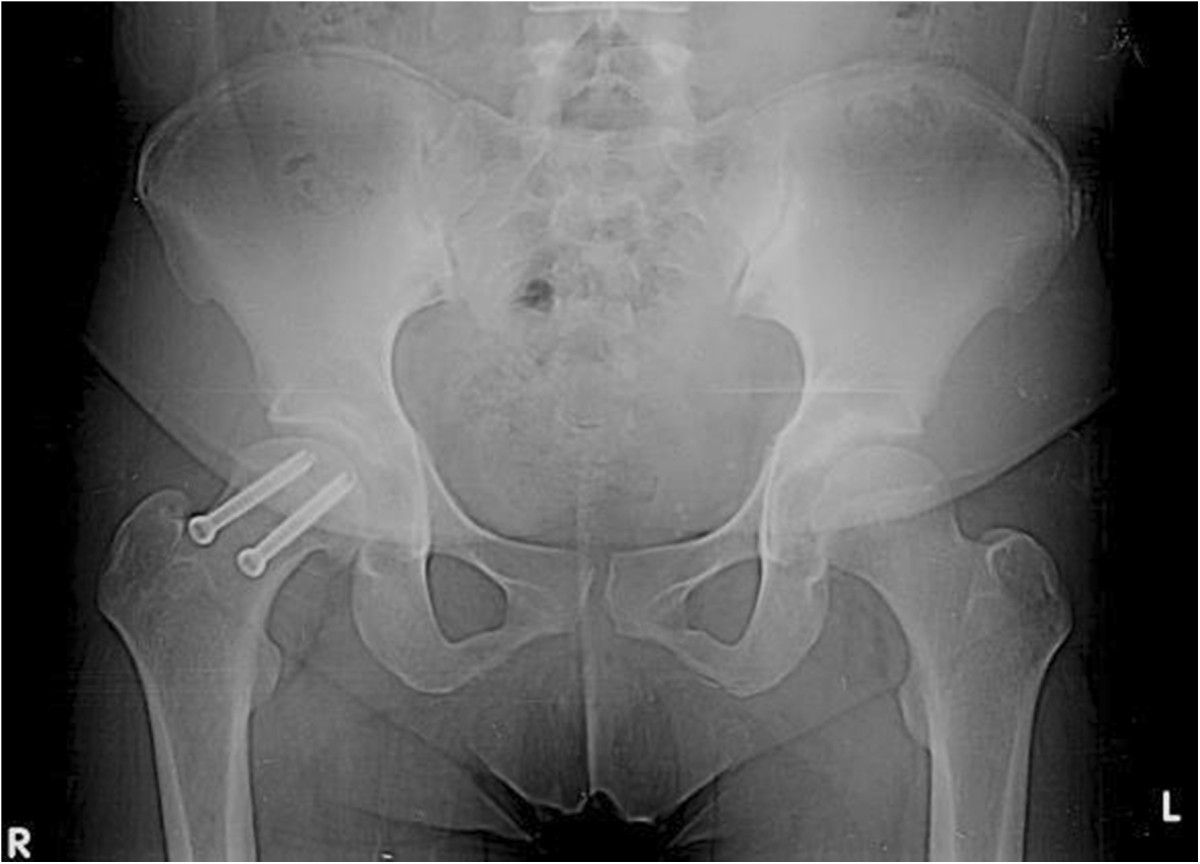
**X-ray control.** On this image obtained 12 months after treatment, appropriate situation of osteosynthetic material can be seen, in addition to reduced physis.

The following hormone profile data were recorded at admission: free triiodothyronine (T3), 1.19pg/ml (normal, 1.71pg/ml to 3.71pg/ml); T3, 0.25ng/ml (normal, 0.58ng/ml to 1.59ng/ml); thyroid-stimulating hormone (TSH), 180.49IU/ml (normal, 0.35IU/ml to 4.94IU/ml); thyroxine (T4), <1.0μg/dl (normal, 4.87μg/dl to 11.72μg/dl); free T4 <0.40ng/dl (normal, 0.70 ng/dl to 1.48ng/dl); parathyroid hormone, 32.9pg/ml (normal, 15pg/ml to 68.3pg/ml); prolactin, 157μg; and cortisol, 14.5μg.Computed tomography (CT) of the sella with contrast enhancement was performed to determine the etiology of hormonal changes, which revealed pituitary hypoplasia secondary to arachnoidocele compression (Figure 4). The CT findings confirmed the diagnosis of secondary hypothyroidism, and treatment with levothyroxine 50μg/day was initiated.Rehabilitation was initiated immediately after surgery, and weight-bearing was deferred for 3 months. The patient’s hip mobility gradually improved. At 4 months after surgery, she achieved free and independent functioning, right hip flexion to 115°, extension to 30°, adduction to 25°, abduction to 50°, internal rotation to 30° and external rotation to 20° (Figure 5).The patient presented adequate thyroid hormone levels in control studies. Follow-up radiographs taken at 12 months of treatment showed appropriate reduction and persistence of open physis with Risser grade IV, yet reduction was seen in comparison to the initial X-rays (Figure 6).

## Discussion

SCFE is the most common hip disorder affecting the adolescent population, usually when they are between 8 and 15 years old [1, 2]. The prevalence of SCFE is 10.8 per 100,000 children [4]. SCFE is known to affect boys more often than girls. Also, it occurs more frequently in African Americans and Pacific Islanders, possibly due to increased body weight in these two subpopulations [5].

The etiology of SCFE is still unclear, and the role of endocrine disruption remains controversial. The authors of some reports have indicated that a deficiency of sex hormones in relation to growth hormones can result in instability of the growth plate [6–8].

Loder *et al.* examined 85 patients with endocrine and SCFE disorders and found that 40% had hypothyroidism, 25% had growth hormone deficiency and 35% had other conditions, such as panhypopituitarism and hyperparathyroidism [9]. This is why, in the clinical examination of our patient, we did a hormone profile to identify a possible etiology.

Thyroid and growth hormones are the most commonly affected hormones. Both are necessary for growth and maturation of the cartilage, with subsequent calcification and replacement by mineralized osteoid. Collectively, this process causes degeneration and proliferation of chondrocytes in the growth plate and subsequent calcification and ossification of the matrix. In patients with hypothyroidism, there is an increase in chondrocyte degradation and mineralization of the matrix is improved; however, ossification of the mineralized matrix is inhibited [3, 10, 11].

Thyroid hormone deficiency can play a significant role in reducing the stability of the growth plate and then contribute to the pathogenesis of the proximal femoral glide [12, 13]. In examining patients with thyroid hormone disorders, it is important to distinguish primary from secondary hypothyroidism. The most useful approach is to determine TSH levels, because elevation is the most sensitive indicator of primary hypothyroidism [13], as evidenced by our case. Our patient had low TSH and high T3 levels, indicating secondary hypothyroidism, which was confirmed by CT scans showing arachnoidocele with compression of the pituitary gland.

Therefore, hypothyroidism must be a diagnosis of exclusion and should be considered in patients who are of relatively low height for their age. Furthermore, hypogonadism must be taken into account in those patients who lack appropriate sexual maturity for their age [3]. Our patient was of short stature and had proper development of sexual characteristics (Tanner 4).

Other associated symptoms may be poorly developed skeletal muscle, which may be associated with radiographic epiphyseal closure of long bones [3] as evidenced by the bone series in our patient, and open physis at the proximal humerus, proximal femur and distal radius. Initial laboratory studies should include follicle-stimulating hormone, luteinizing hormone, testosterone, prolactin and estradiol, and they must include thyroid function tests [9].

Patients with unilateral SCFE should be carefully monitored to detect changes in an asymptomatic or unaffected hip to avoid delays in diagnosis [2]. We have followed our patient for 12 months, during which time no slip in the contralateral proximal femoral physis has been observed.

## Conclusions

There are very few case reports in the worldwide literature of adult patients who presented with SCFE. It should be emphasized that all of the published cases were associated with different endocrine disorders, either pituitary diseases (generally tumoral) or, more rarely, hypothyroidism, and all were of primary origin. To our knowledge, there has been no case report published to date describing a diagnosis of hypothyroidism secondary to arachnoidocele compression.

## Consent

Written informed consent was obtained from the patient for publication of this case report and any accompanying images. A copy of the written consent is available for review by the Editor-in-Chief of this journal.

## Authors’ original submitted files for images

Below are the links to the authors’ original submitted files for images. Authors’ original file for figure 1
Authors’ original file for figure 2
Authors’ original file for figure 3
Authors’ original file for figure 4
Authors’ original file for figure 5
Authors’ original file for figure 6

## Abbreviations

SCFE: Slipped capital femoral epiphysis.
