# Supplementary figures and images for: Slipped capital femoral epiphysis and hypothyroidism in a young adult: a case report
Source: J Med Case Rep. 2014 Oct 10;8(1):336. doi: 10.1186/1752-1947-8-336 (PMC5011917; doi:10.1186/1752-1947-8-336)

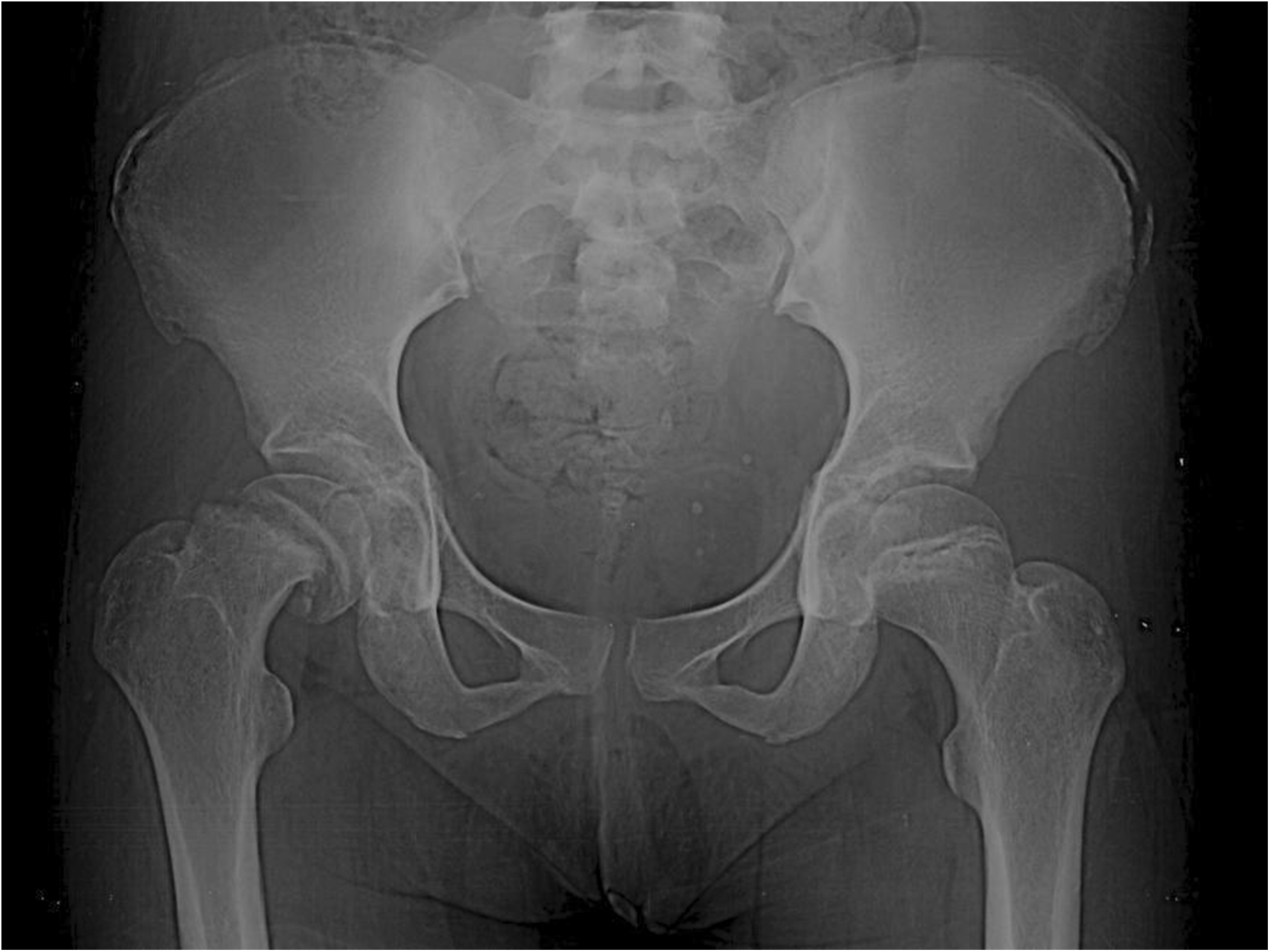

Supplement: Supplementary file 1 — Authors’ original file for figure 1 [file 13256_2014_3136_MOESM1_ESM.tif]

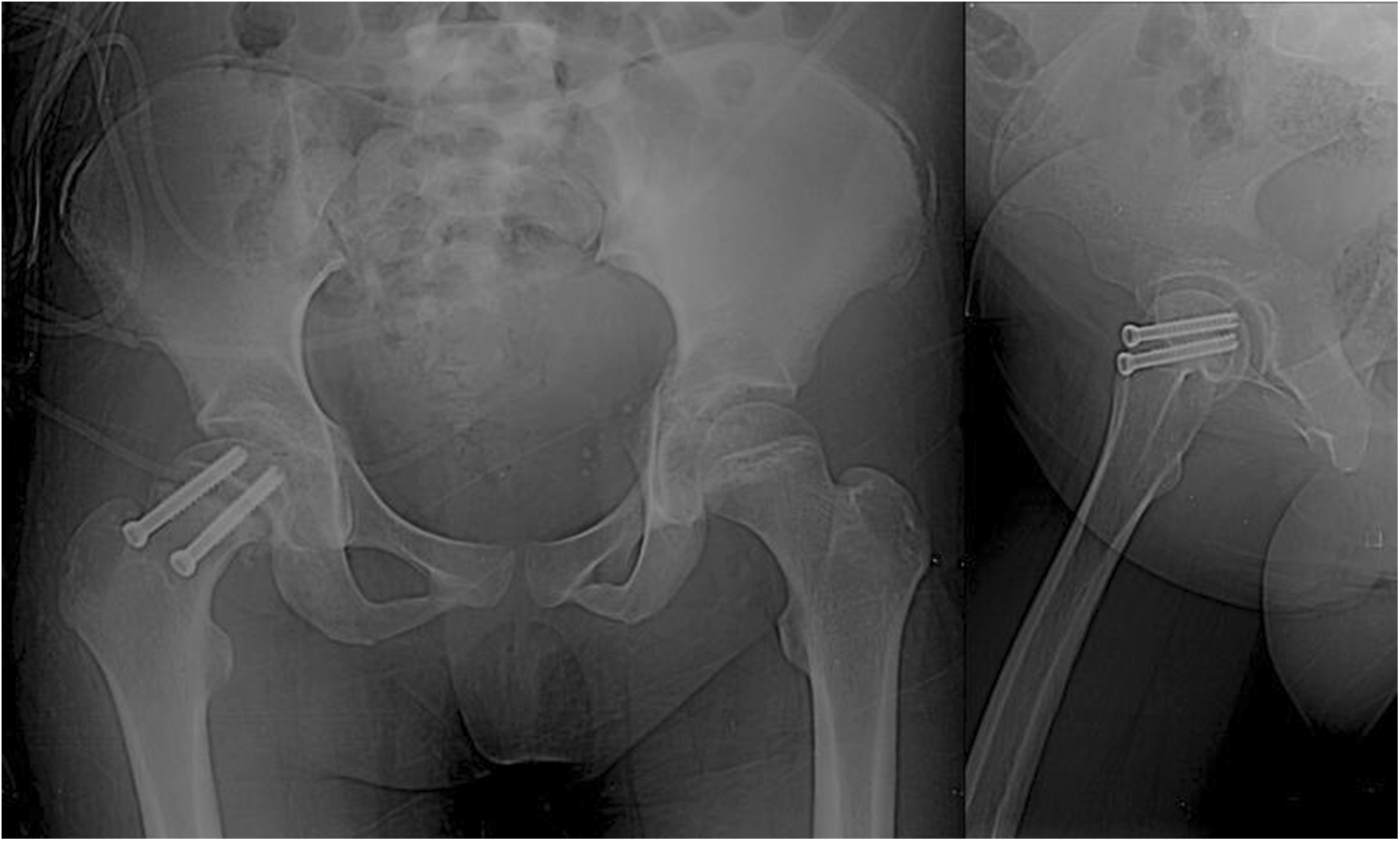

Supplement: Supplementary file 2 — Authors’ original file for figure 2 [file 13256_2014_3136_MOESM2_ESM.tif]

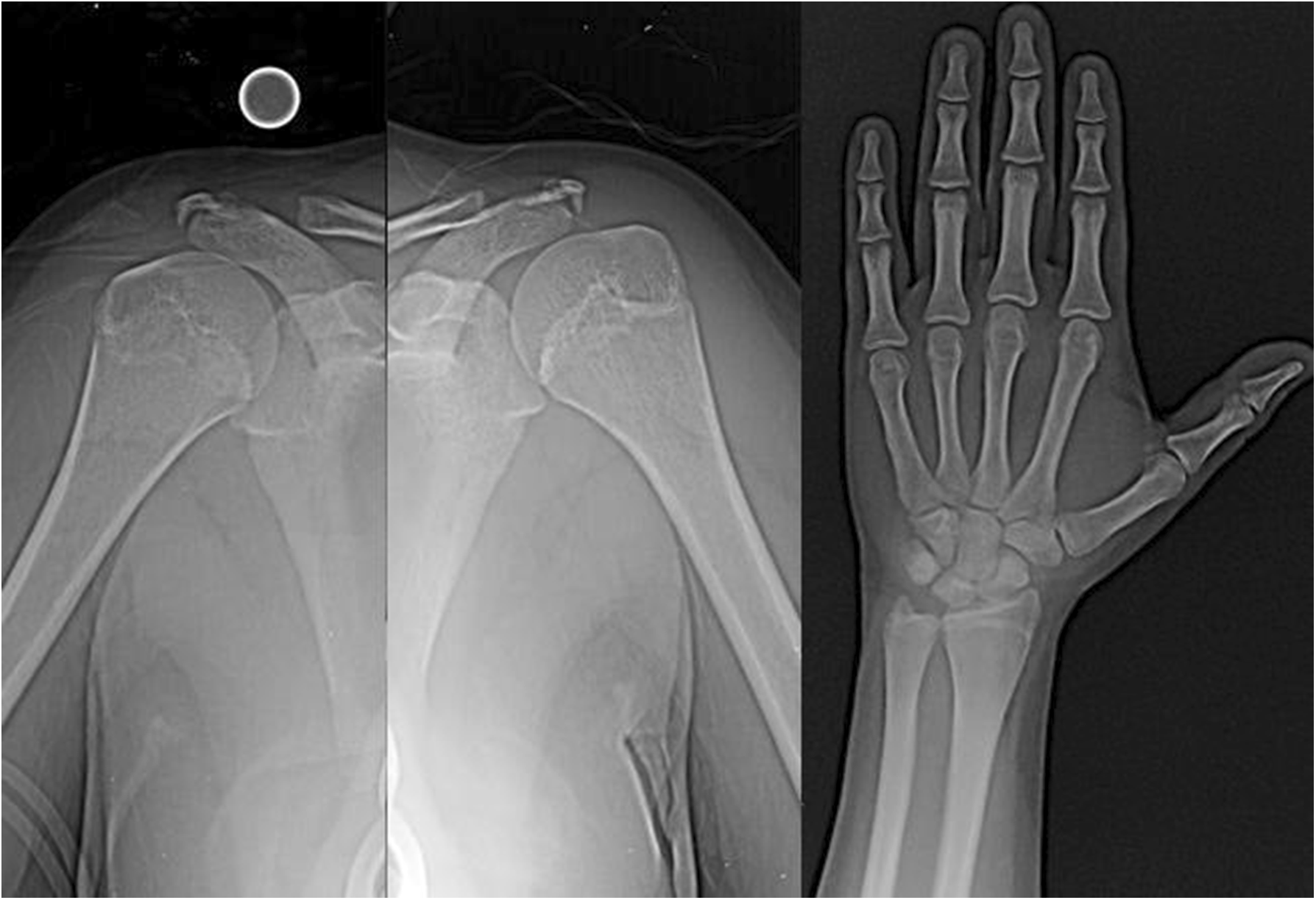

Supplement: Supplementary file 3 — Authors’ original file for figure 3 [file 13256_2014_3136_MOESM3_ESM.tif]

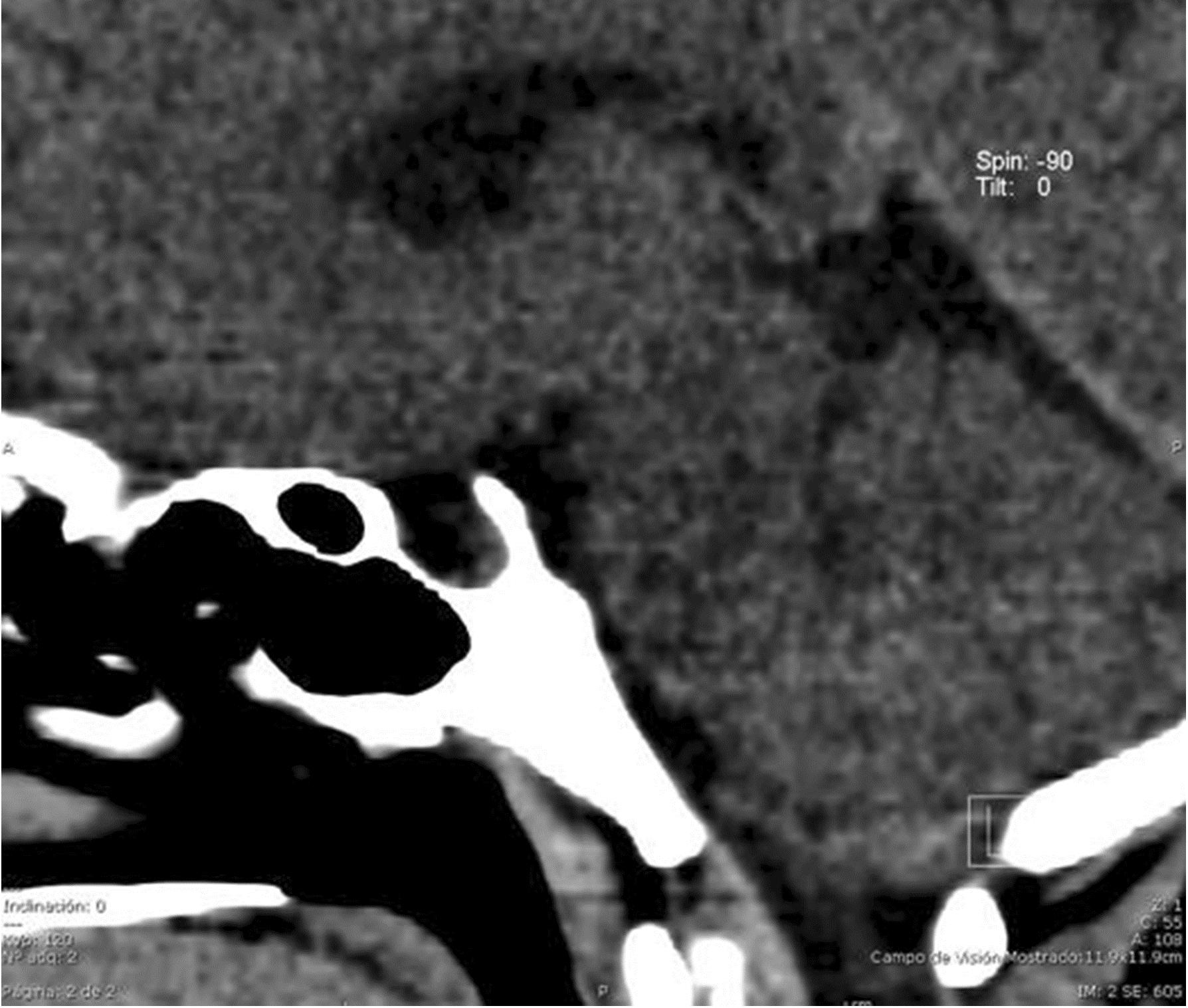

Supplement: Supplementary file 4 — Authors’ original file for figure 4 [file 13256_2014_3136_MOESM4_ESM.tiff]

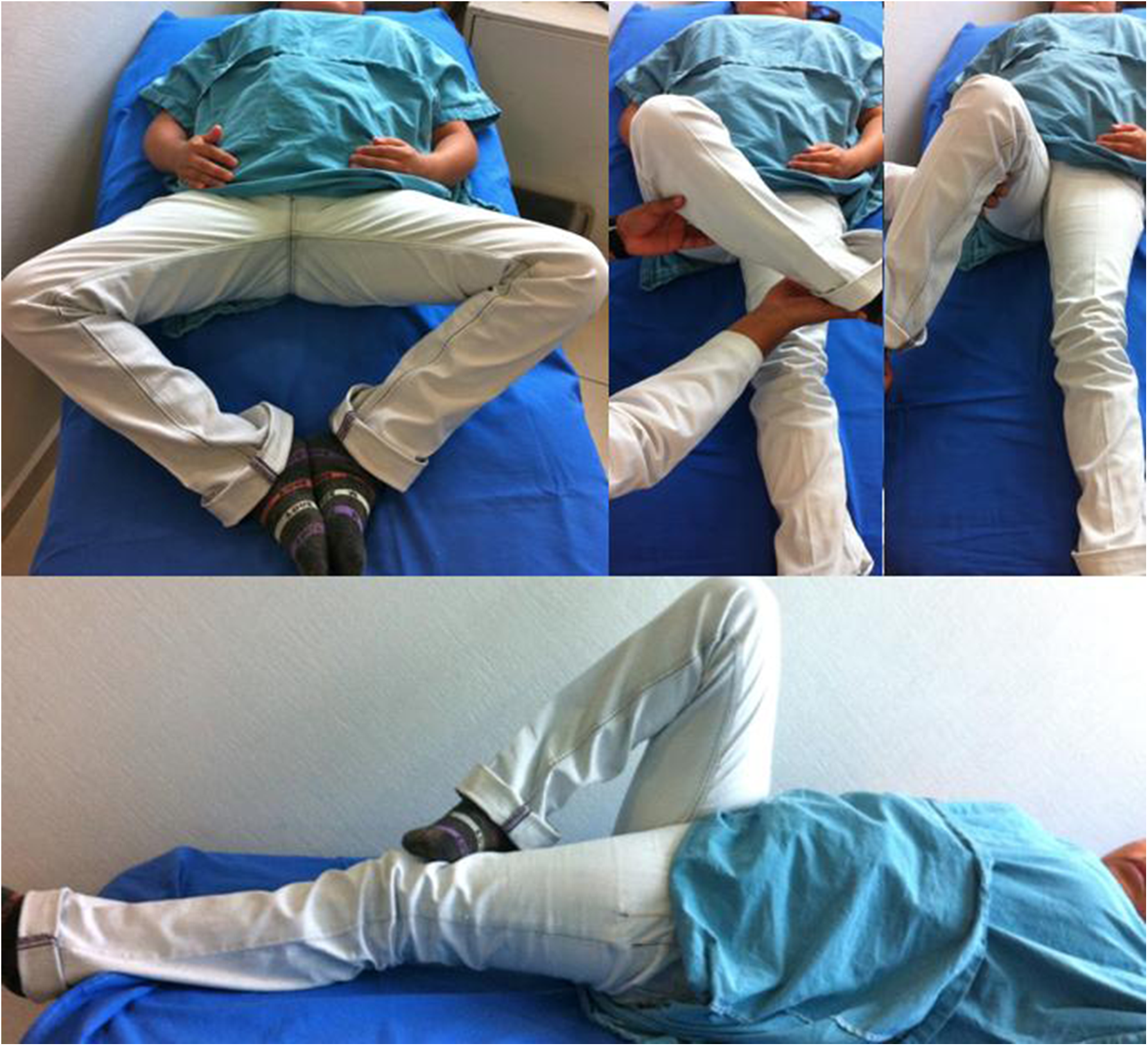

Supplement: Supplementary file 5 — Authors’ original file for figure 5 [file 13256_2014_3136_MOESM5_ESM.tif]

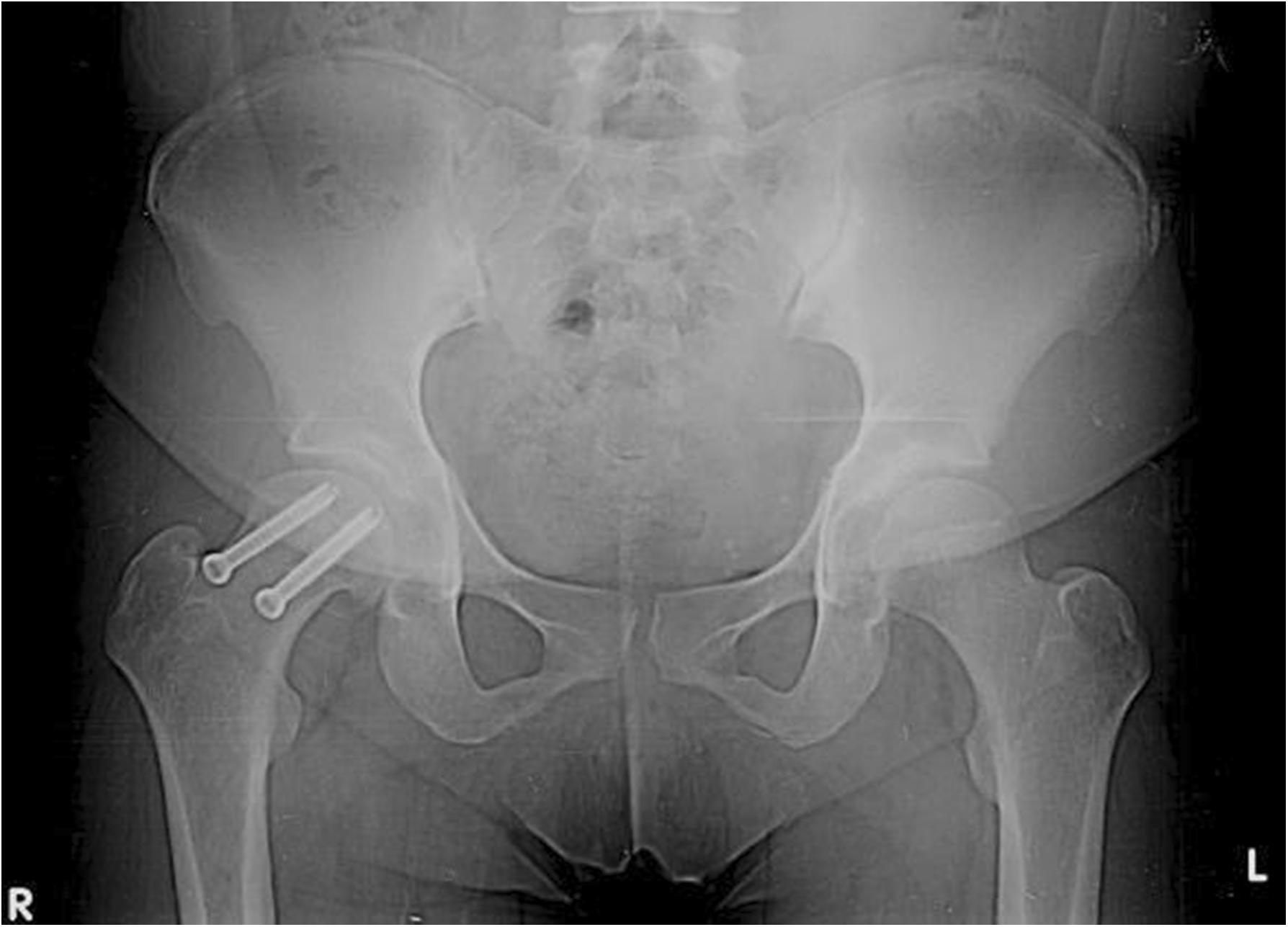

Supplement: Supplementary file 6 — Authors’ original file for figure 6 [file 13256_2014_3136_MOESM6_ESM.tif]
